# Usefulness of the Neutrophil-to-Lymphocyte Ratio as a Predictor of Pneumonia and Urinary Tract Infection Within the First Week After Acute Ischemic Stroke

**DOI:** 10.3389/fneur.2021.671739

**Published:** 2021-05-13

**Authors:** Robin Gens, Anissa Ourtani, Aurelie De Vos, Jacques De Keyser, Sylvie De Raedt

**Affiliations:** ^1^Vrije Universiteit Brussel (VUB), Universitair Ziekenhuis Brussel (UZ Brussel), Department of Neurology/Center for Neurosciences, Brussels, Belgium; ^2^Centre Hospitalier Universitaire Brugmann (CHU Brugmann), Department of Neurology, Brussels, Belgium; ^3^Department of Neurology, Sint-Maria Halle, Halle, Belgium; ^4^Faculty of Medicine and Pharmacy, Vrije Universiteit Brussel, Brussels, Belgium

**Keywords:** acute ischemic stroke, post-stroke pneumonia, post-stroke urinary tract infection, post-stroke infections, neutrophil-to-lymphocyte ratio

## Abstract

**Background:** A high Neutrophil-to-Lymphocyte ratio (NLR) in patients with acute ischemic stroke (AIS) has been associated with post-stroke infections, but it's role as an early predictive biomarker for post-stroke pneumonia (PSP) and urinary tract infection (UTI) is not clear.

**Aim:** To investigate the usefulness of NLR obtained within 24 h after AIS for predicting PSP and UTI in the first week.

**Methods:** Clinical and laboratory data were retrieved from the University Hospital Brussels stroke database/electronic record system. Patients were divided into those who developed PSP or UTI within the first week after stroke onset and those who didn't. Receiver operating characteristics (ROC) curves and logistic regression analysis were used to identify independent predictors.

**Results:** Five hundred and fourteen patients were included, of which 15.4% (*n* = 79) developed PSP and 22% (*n* = 115) UTI. In univariate analysis, NLR was significantly higher in patients who developed PSP (4.1 vs. 2.8, *p* < 0.001) but not in those who developed UTI (3.3 vs. 2.9, *p* = 0.074). Multiple logistic regression analysis for PSP showed that NLR, male gender, dysphagia, and stroke severity measured by the National Institutes of Health Stroke Scale (NIHSS), were independent predictors of PSP. For NLR alone, the area under the curve (AUC) in the ROC curve was 0.66 (95% CI = 0.59–0.73). When combining NLR ≥ 4.7 with age >75 years, male gender, NIHSS > 7, and dysphagia, the AUC increased to 0.84 (95% CI = 0.79–0.89).

**Conclusion:** The NLR within 24 h after AIS appears to have no predictive value for post-stroke UTI, and is only a weak predictor for identifying patients at high risk for PSP. Its predictive value for PSP appears to be much stronger when incorporated in a prediction model including age, gender, NIHSS score, and dysphagia.

## Introduction

Pneumonia and urinary tract infections (UTI) are the most common infectious complications after acute ischemic stroke (AIS), with an incidence of 12 and 16%, respectively (1). Post-stroke infections have been associated with poor outcome and mortality (2, 3). Therefore, there is an interest in finding early predictors of these post-stroke infections, which may help to select high-risk patients to start interventions in time. Most prediction scoring models for post-stroke pneumonia (PSP) are based on clinical features including age, gender, stroke severity measured by the National Institutes of Health Stroke Scale (NIHSS) (4) and the presence of dysphagia (5–9). A recent meta-analysis showed that age, female gender and post-void residual volume >100 ml were predictors of post-stroke UTI (10).

Next to clinical factors, a number of inflammatory parameters including C-reactive protein (CRP), white blood cell count, procalcitonin and copeptin (11), interleukin-13 and interferon-γ (12), elevated monocyte count and interleukin-10 (13), and high circulating natural killer cell count within the first hours after stroke followed by a drop in all lymphocyte subsets (14) have been associated with post-stroke infections. However, it is unclear how these parameters should be applied in clinical practice.

A biomarker, which has gained interest over the last years, is the Neutrophil-to-Lymphocyte Ratio (NLR). It is a marker of inflammation that is simply calculated from blood cell counts obtained on admission in every AIS patient. Nam et al. (15) found that a higher NLR in patients with AIS who were admitted within 7 days of symptoms onset independently predicted PSP during that 7-day period. Wang et al. (16) found that the NRL at multiple time points with a peak at 36 h after stroke onset was independently associated with PSP but not with UTI. The NLR on admission was not used separately in their study. Three other studies in patients with AIS in whom blood was collected within 24 h of symptom onset did not discriminate between PSP, UTI, and other infections. Two of them found that a higher NLR was independently associated with post stroke infections (17, 18), whereas the third study could not confirm this association (19).

Since most of these infections already manifest within the first days after AIS, we wanted to investigate the predictive value of NLR obtained on admission within 24 h after stroke onset for PSP and UTI separately.

## Materials and Methods

### Patients and Assessment Procedures

**Figure 1:**
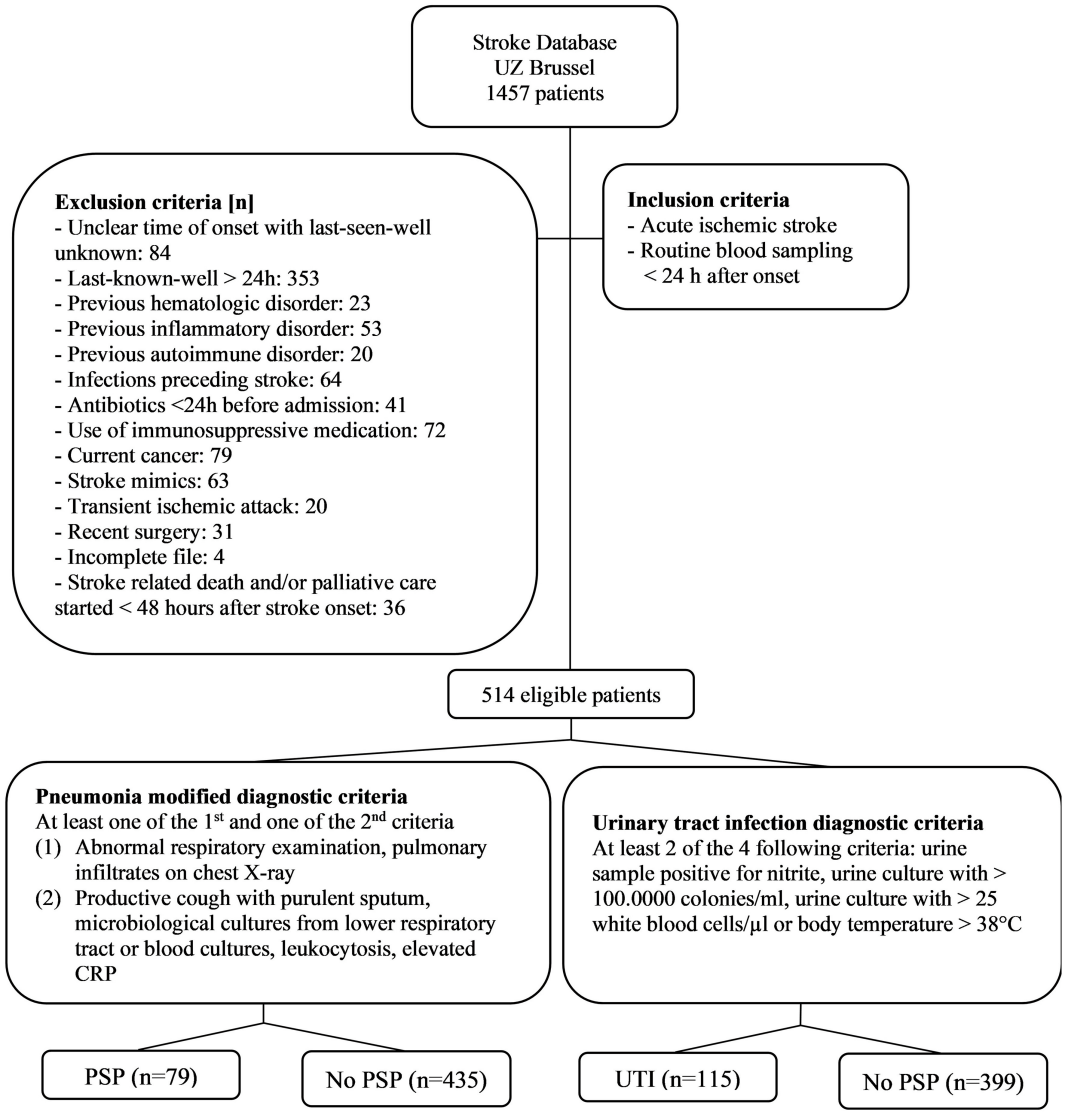
Study population flowchart. CRP, C-reactive protein; PSP, post-stroke pneumonia.

We extracted the data of 1,457 patients admitted to the Stroke Unit of the University Hospital Brussels (Belgium), which were prospectively collected in a database over a 6-year period. We included all patients with AIS, who had routine blood sampling within 24 h after stroke onset. AIS was defined as “a sudden onset of loss of global or focal cerebral function” (20) caused by brain ischemia of any origin, confirmed on cerebral computed tomography, or magnetic resonance imaging. Exclusion criteria were previous hematologic, inflammatory or autoimmune disorders, current cancer, infections preceding stroke, use of antibiotics <24 h before admission, use of immunosuppressants on admission, recent surgery, and stroke related death and/or palliative care started <48 h after stroke onset. A study population flowchart is shown in Figure 1. Demographic data (age, gender), medical history, use of beta-blockers prior to admission, pre-stroke modified Rankin Scale (mRS), NIHSS on admission, level of consciousness (LOC, determined by NIHSS subitem 1a) and information concerning intravenous thrombolysis (IVT) and endovascular therapy (EVT) were retrieved from the database. Dysphagia objectified by a professional speech therapist, nasogastric tube feeding, urinary catheter placement, and results of baseline blood measures (absolute neutrophil count, absolute lymphocyte count and CRP) were retrieved from the electronic record system.

### Standard Protocol Approval

The study protocol was approved by the Ethics Committee of the University Hospital of Brussels (reference number B.U.N. 143201733949).

### Neutrophil-to-Lymphocyte Ratio

The NLR was defined as the ratio of the absolute neutrophil count to the absolute lymphocyte count, which were counted in the peripheral blood sample on admission by use of fluorescent flowcytometric measurements (CELL-DYN Sapphire, Abbott Diagnostics, Abbott Park, IL) (14, 21).

### Post-stroke Pneumonia

PSP during the first week after stroke onset was retrospectively diagnosed using Modified criteria of the US Center for Disease Control and Prevention: “at least one of the former and one of the latter criteria fulfilled: (A) abnormal respiratory examination, pulmonary infiltrates on chest x-rays; (B) productive cough with purulent sputum, microbiological cultures from lower respiratory tract or blood cultures, leukocytosis, elevated CRP” (14, 22).

### Post-stroke UTI

UTI during the first week after stroke onset was retrospectively diagnosed and defined as having at least 2 of the 4 following criteria: urine sample positive for nitrite, urine culture with >100.0000 colonies/ml, urine culture with >25 white blood cells/μl or body temperature >38°C (22).

### Statistics

Statistical analyses were performed using SPSS version 27.0 software package. Patients were divided into those who developed PSP/UTI and those who didn't. Normality was checked by using the Kolmogorov-Smirnov test and visual interpretation of histograms and Q-Q plots. Skewed variables were log-transformed to reach normality. Differences were detected using the Independent-Samples Student *T*-test (with back-transformation of the results, if applicable) and the Mann-Whitney U-test for continuous variables. The χ2- or Fisher Exact-test were used for categorical variables. Age and NIHSS on admission were dichotomized by using the values of the 50% percentile as cut-off. For NLR, the 75% percentile was used. Variables of clinical interest were enrolled in multiple logistic regression analysis (MLRA). The stepwise Backward Wald method and ROC curves were used to identify independent predictors. Variables most accessible on admission were combined to create a prediction model.

## Results

### Baseline Characteristics

Five hundred and fourteen patients met the selection criteria, of whom 15% (*n* = 79) developed PSP and 22% (*n* = 115) developed UTI (Figure 1). Table 1 presents the baseline characteristics of patients with PSP vs. without PSP, and of patients with post-stroke UTI vs. without post-stroke UTI.

**Table 1:** Baseline characteristics of study population (*n* = 514).

**Variables**	**Post-stroke pneumonia**			**Post-stroke UTI**		
	**PSP (*n* = 79)**	**No PSP (*n* = 435)**	***p*-value**	**UTI (*n* = 115)**	**No UTI (*n* = 399)**	***p*-value**
Age, years^a^	79 (69–86)	74 (62–83)	0.005	79 (74–87)	72 (61–82)	<0.001
Gender, male^b^	54 (68.4)	223 (51.3)	0.005	32 (27.8)	245 (61.4)	<0.001
Known AHT^b^	62 (78.5)	313 (72.0)	0.230	89 (77.4)	286 (71.7)	0.224
Use of β-blockers^b^	36 (45.6)	166 (38.2)	0.215	50 (43.5)	152 (38.1)	0.298
Known DM^b^	19 (24.1)	86 (19.8)	0.385	25 (21.7)	80 (20.1)	0.692
NIHSS^a^	16 (8–21)	5 (2–12)	<0.001	10 (5–18)	5 (2–14)	<0.001
Altered LOC (NIHSS subitem 1a > 0)^b^	23 (29.1)	27 (6.6)	<0.001	18 (16.2)	32 (8.4)	0.017
Dysphagia^b^	47 (59.5)	90 (20.7)	<0.001	47 (41.6)	90 (23.1)	<0.001
IVT^b^	39 (49.4)	120 (27.6)	<0.001	34 (29.6)	125 (31.3)	0.719
EVT^b^	4 (6.3)	24 (5.5)	0.789	5 (5.1)	23 (5.8)	0.783
Tube feeding^b^	42 (53.2)	40 (9.2)	<0.001	31 (27.2)	51 (12.8)	<0.001
Urinary catheter	27 (34.2)	63 (14.5)	<0.001	33 (28.9)	57 (14.3)	<0.001
#Lymphocytes (/mm^3^)^c^	1598 ± 1.7	1869 ± 1.6	0.344	1746 ± 1.6	1848 ± 1.6	0.245
#Neutrophils (/mm^3^)^c^	6503 ± 1.55	5251 ± 1.53	<0.001	5796 ± 1.6	5319± 1.5	0.064
NLR^c^	4.1 ± 2.1	2.8 ± 1.9	<0.001	3.3 ± 2.2	2.9 ± 1.9	0.074
CRP (mg/l)^a^	3.2 (1.6–11.1)	2.6 (1.2–5.8)	0.035	2.9 (1.2–6.3)	2.9 (1.3–6.4)	0.703

*Results are expressed as mean ± standard deviation (SD), median (interquartile range (IQR)) or n (%) when appropriate. PSP, post-stroke pneumonia; NIHSS, National Institutes of Health Stroke Scale; AHT, arterial hypertension; DM, diabetes mellitus; IVT, intravenous thrombolysis; EVT, endovascular therapy; NLR, Neutrophil-to-Lymphocyte Ratio; CRP, C-reactive protein*.

a *Mann-Whitney U-test*.

b *χ2-test*.

c *Independent-Samples Student t-test*.

### Post-stroke Pneumonia

In univariate analysis, age, male gender, NIHSS, altered LOC, treatment with IVT, dysphagia, tube feeding and urinary catheter placement were associated with PSP (p < 0.05). Patients who developed PSP had significantly lower lymphocyte counts on admission. CRP, neutrophil count, and NLR within 24 h after stroke onset were significantly higher in patients with vs. without PSP. The NLR was not significantly different between patients who developed PSP during the first 3 days (71% of PSP cases) of admission and those who developed PSP between day 4 and 7 (29% of cases) of admission (4.29 ± 2.07 vs. 3.59 ± 1.99 respectively, p = 0.320). Of all patients, 145 patients were discharged before day 7. The mean length of their hospital stay was 4.75 ± 1.2 days, which exceeded the mean time to onset of PSP of 2.9 ± 1.7 days for the entire study population. The mean time to event did not significantly differ between patients who had a hospital stay of 7 days or more vs. those who were discharged before day 7 (3.0 ± 1.8 vs. 2.4 ± 1.2 days, p = 0.301).

### Post-stroke UTI

In univariate analysis, age, female gender, pre-stroke mRS, NIHSS, dysphagia, tube feeding, altered LOC, and urinary catheter placement were associated with post-stroke UTI (p < 0.05). NLR within 24 h after stroke onset was not significantly higher in patients with post-stroke UTI compared to patients without post-stroke UTI. The NLR was not predictive in both patients discharged before day 7 and those who stayed for 7 days or more.

### Multiple Logistic Regression

Since NLR was not significant in univariate analysis for UTI, we opted to perform multivariate analysis for PSP only. The following variables were enrolled in MLRA: age, gender, smoking, chronic obstructive pulmonary disease (COPD), diabetes mellitus (DM), NIHSS, LOC, dysphagia, and NLR. The results indicated that NLR, next to age, male gender, NIHSS on admission, and dysphagia, was an independent predictor of PSP (Table 2). To create a more easy-to-use prediction model, we dichotomized “NIHSS on admission” and “age” by using the 50% percentile values as cut-offs, which were >7 and >75 years, respectively. The cut-off for NLR was determined by the 75% percentile value, which was ≥4.7. Based on the results of the first multivariate analysis and the clinical usefulness of the variables, we repeated MLRA using the following variables: age > 75 years, male gender, dysphagia, NIHSS > 7 and NLR ≥ 4.7, which shows a significant predictive value for each of these variables when using this model (Table 2).

**Table 2:** Stepwise MLRA for PSP (model 1a and 1b).

	**Variables**	**OR**	**95% CI**	**p-value**
Including continuous variables	Age	1.03	1.00–1.05	0.047
	Male gender	4.40	2.27–8.54	<0.001
	Dysphagia	5.20	2.71–9.97	<0.001
	NIHSS	1.08	1.04–1.13	<0.001
	NLR	1.12	1.04–1.21	0.003
Prediction model	Age > 75 years	2.45	1.31–4.58	0.005
	Male gender	4.14	2.16–7.93	<0.001
	Dysphagia	6.40	3.36–12.20	<0.001
	NIHSS > 7	2.54	1.29–5.01	0.007
	NLR ≥ 4.7	2.89	1.60–5.22	<0.001

*MLRA, multiple logistic regression analysis; PSP, post-stroke pneumonia; OR, odds ratio; CI, confidence interval; NIHSS, National Institutes of Health Stroke Scale; NLR, Neutrophil-to-Lymphocyte ratio*.

### ROC Curve Analyses

**Figure 2:**
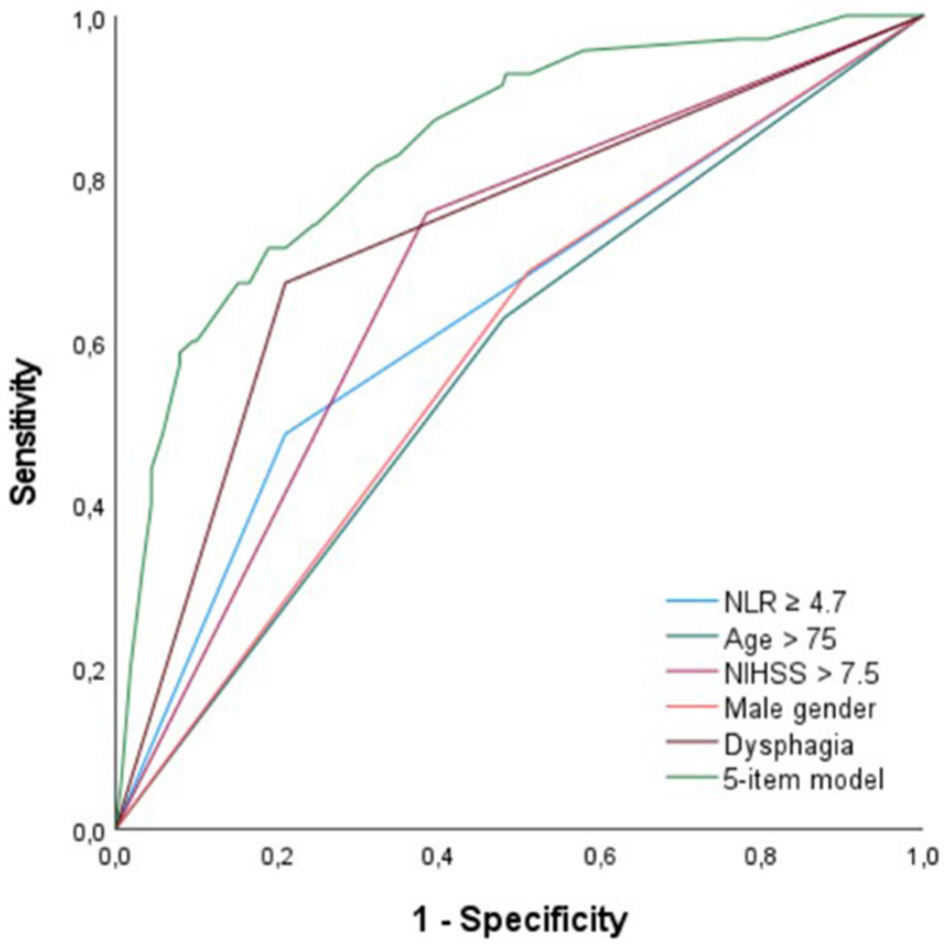
ROC curve analysis for NLR ≥ 4.7, age > 75 years, NIHSS > 7, male gender, dysphagia, and a 5-item prediction model (NLR ≥ 4.7, age > 75 years, dysphagia, NIHSS > 7, male gender). ROC, receiver operating characteristics; NLR, neutrophil-to-lymphocyte ratio; NIHSS, National Institutes of Health Stroke Scale.

For NLR, age, NIHSS, and male gender, AUC was to 0.66 (95% CI = 0.59–0.73), 0.60 (95% CI = 0.53–0.66), 0.75 (95% CI = 0.68–0.81) and 0.59 (95% CI = 0.52–0.66), respectively (see Figure 2). For the dichotomized variables, NLR ≥ 4.7, age > 75 years, and NIHSS > 7, AUC was 0.64 (95% CI = 0.56–0.71), 0.58 (95% CI = 0.50–0.65), and 0.68 (95% CI = 0.62–0.75), respectively (Figure 2). For a 5-item prediction model, which combines age > 75, male gender, dysphagia, NIHSS > 7, and NLR ≥ 4.7, AUC was 0.84 (95% CI = 0.79–0.89) (Figure 2).

## Discussion

Previous studies have shown that NLR is a predictor of poor functional outcome and mortality after AIS, but the underlying mechanisms remain unclear (20, 23–26). Two studies found a link between the NLR and post-stroke infections but they lack information about the location of the infection (17, 18). A study by Nam et al. (15) found that a NLR cut-off value >2.43, which was based on the median of their cohort, was an independent predictor of PSP. However, NLR was determined within 7 days of stroke onset instead of 24 h. In another study, a higher NLR at different time points post-stroke, with a peak value at 36 h, has also been associated with post-stroke infection, and more specific PSP (16). A study of van Gemmeren did not show an independent predictive value of the 24 h NLR for PSP, but because numbers were small the study was likely underpowered to detect such an effect (19).

Our results provide additional evidence for NLR as a significant and independent predictor for PSP, although, its predictive value appears to be quite weak. ROC curve analysis of NLR alone showed an AUC of 0.66 (95% CI = 0.59–0.73). This could be explained by the fact that immunological changes are only one of the mechanisms leading to PSP. Our results further showed that age, male gender, dysphagia, and stroke severity (NIHSS) were, albeit also weak, independent predictors of PSP, which is in line with previous studies (5–9, 27–29). Because of its rather low predictive value for PSP, we reperformed MLRA with only dichotomized variables, to make it more clinically useful. Based on the results of our first multivariate analysis and the immediate availability upon admission of the enrolled clinical variables, we created a 5-item prediction model using NLR ≥ 4.7, age > 75 years, male gender, dysphagia, and NIHSS > 7. In this model, the AUC increased to 0.84 (95% CI = 0.79–0.89), indicating that NLR is especially useful in predicting PSP when incorporated into a model with these four clinical predictive factors.

The NLR was not significantly different in patients who developed PSP within 3 days of admission and those who developed PSP during day 4–7 after admission, suggesting that a high admission NLR is not solely due to an inflammatory response caused by aspiration, or a pneumonia, that was already started on admission.

Our study found that NLR within 24 h after stroke onset was not a significant predictor of UTI. This confirms the findings of Wang et al. (16) who also did not find a significantly higher NLR in patients with post-stroke UTI, although, they did not use the NLR on admission. A plausible explanation why NLR is predictive for PSP but not for UTI, is that the underlying mechanisms of these infections are at least partially different. After AIS, neutrophil counts increase and lymphocyte counts decrease (28, 30, 31) as part of the post-stroke immunodepression phenomenon, activated by the sympathetic nervous system and hypothalamic-pituitary-adrenal axis (30, 32). This may be a mechanism to prevent further damage by reducing local brain inflammation. The role of neutrophils and lymphocytes seems to be dual, with both beneficial and harmful effects (3, 14, 31, 33). The NLR could be used to estimate the degree to which this post-stroke immunodepression occurs, with a higher NLR suggestive of a more pronounced immunodepression. Since both NLR and pneumonia have been associated with poor prognosis after ischemic stroke (20, 24, 34–36), we hypothesize that a higher degree of immunodepression makes patients more susceptible to systemic infections, such as pneumonia, leading to a worse outcome. Preclinical evidence shows that mice subjected to ischemic stroke were more susceptible to spontaneous bacteriemia and pneumonia compared to mice who underwent sham procedure (37). An explanation might be that the post-stroke immunodepression phenomenon favors bacterial translocation and dissemination of commensal bacteria from the host gut microbiota, leading to systemic infections (38). Whereas, these mechanisms might contribute to PSP, the occurrence of post-stroke UTI seems to rather depend on other factors. Urinary tract infections, which can be seen as rather local than systemic infections, seem to be mainly explained by mechanical factors such as bladder dysfunction causing urinary retention (39), use of urinary catheter (29, 40) and the presence of a short urethra (female predominance). In addition, they are less clearly associated with worse prognosis after ischemic stroke, since although preventive antibiotics reduced UTI frequency in the PASS-study, no effect was seen on outcome (36).

It has been hypothesized that sympathetic nervous system activation might be one of the underlying mechanisms of post-stroke immunodepression, and that therefore beta-blockers might theoretically prevent post-stroke infections (32). In mice, blockade of the sympathetic pathways by beta-blockers reduced post-stroke infections and improved stroke outcome (41). However, in human studies, results have been conflicting. Sykora et al. (42) reported that pre-stroke and on-stroke beta-blocker treatment reduced PSP frequency. On the other hand, Maier and coworkers reported that beta-blocker exposure had no effect on PSP frequency, but that it reduced UTI rates (43, 44). Dromerick et al. (45) found the use of beta-blockers to be a predictor of post-stroke UTI. In our study, we did not find an association between beta-blocker use prior to AIS and PSP or UTI.

There are some limitations to this study. First, although data were gathered prospectively, the diagnosis of PSP and UTI was checked retrospectively, which could have caused some diagnostic errors. By using the modified CDC criteria for retrospective diagnosis of pneumonia, a positive chest x-ray was not necessary to reach diagnostic criteria. Therefore, diagnosis could also be made based on clinical features only, which might have decreased diagnostic accuracy. Second, the NLR was only investigated for its predictive role regarding PSP/UTI. It is possible that patients developed other infectious or inflammatory complications that might have influenced NLR. Third, we did not intend to exclude patients discharged before day 7, as we wanted to explore the role of NLR and the subsequent combined model in a situation consistent with real-life in which we do not know in advance how long patients will stay. We may have missed a number of cases with PSP and UTI in patients who were discharged before day 7. However, because the majority of patients (72%) was hospitalized for 7 days or more, it is unlikely that this will affect our main conclusions. In addition, for UTI, the NLR was not predictive in both patients discharged before day 7 and those who stayed for 7 days or more. For PSP, we found that the mean length of hospital stay for those discharged before day 7 exceeded the mean time to onset of PSP, which is usually within the first 2 to 3 days after stroke onset.

Prospective studies are required to investigate whether our proposed prediction model, which incorporates NLR, too can, with a high degree of certainty, identify patients prone to develop PSP, who may therefore be candidates for prophylactic measures. Prophylactic antibiotic treatment significantly decreases overall post-stroke infection rate, but its effect on reducing the incidence of PSP has not been established (46). Identifying patients at risk will lead to a better selection of patients who could benefit from this kind of treatment. In addition, new therapeutic approaches other than prophylactic antibiotic administration, such as treatment of the underlying mechanisms of post-stroke immunodepression, should be considered (32).

## Data Availability Statement

The raw data supporting the conclusions of this article will be made available by the authors, without undue reservation.

## Ethics Statement

The studies involving human participants were reviewed and approved by Commissie Medische Ethiek UZ Brussel (reference number B.U.N. 143201733949). Written informed consent for participation was not required for this study in accordance with the national legislation and the institutional requirements.

## Author Contributions

RG: design and conceptualized study, major role in the acquisition of data, analyzed the data, and drafted the manuscript for intellectual content. AO: design and conceptualized study, major role in the acquisition of data, analyzed the data, and drafted the manuscript for intellectual content. AD: major role in the acquisition of data and revised the manuscript for intellectual content. JD: revised the manuscript for intellectual content. SD: design and conceptualized study, major role in the acquisition of data, and drafted the manuscript for intellectual content. All authors contributed to the article and approved the submitted version.

## Conflict of Interest

The authors declare that the research was conducted in the absence of any commercial or financial relationships that could be construed as a potential conflict of interest.
